# Long noncoding RNA EGFR-AS1 promotes cell growth and metastasis via affecting HuR mediated mRNA stability of EGFR in renal cancer

**DOI:** 10.1038/s41419-019-1331-9

**Published:** 2019-02-15

**Authors:** Anbang Wang, Yi Bao, Zhenjie Wu, Tangliang Zhao, Dong Wang, Jiazi Shi, Bing Liu, Shuhan Sun, Fu Yang, Linhui Wang, Le Qu

**Affiliations:** 1 Department of Urology, Changzheng Hospital, Second Military Medical University, 415 Fengyang Road, 200003 Shanghai, China; 2 Department of Urology, Jinling Hospital, Nanjing University Clinical School of Medicine, 210002 Nanjing, China; 3 Department of Medical Genetics, Second Military Medical University, 200433 Shanghai, China; 4 Shanghai Key Laboratory of Cell Engineering (14DZ2272300), Second Military Medical University, 200433 Shanghai, China

## Abstract

Long noncoding RNAs (lncRNAs) are implicated in renal cell carcinoma (RCC), but remain largely unclear. Using publicly available transcriptome sequencing data from renal cancer (*n* = 703) and integrating bioinformatics analyses, we screened and identified a valuable lncRNA, EGFR-AS1. In our validation cohort (*n* = 204), EGFR-AS1 was significantly upregulated in RCC tissues (*P* *<* 0.001). Gain-of-function and loss-of-function studies showed that EGFR-AS1 promoted cell proliferation and invasion in vitro and in vivo. Based on previous studies and sequence complementarity of EGFR with EGFR-AS1, we demonstrated that EGFR-AS1 directly bound to EGFR mRNA and inhibited its degradation. Furthermore, RNA pull-down and mass spectrometry analyses showed that EGFR-AS1 interacted with HuR, which was responsible for the mRNA stability of EGFR. Multivariate analysis suggested that higher EGFR-AS1 expression predicted a poor prognosis in RCC patients (high vs low: *P* = 0.018, HR = 2.204, 95% CI: 1.145–4.241). In conclusion, EGFR-AS1 enhances the malignant phenotype of RCC cells by enhancing HuR-mediated mRNA stability of EGFR. Our data also provide biological rationales for EGFR-AS1 as a prognostic biomarker and a potential therapeutic target for RCC.

## Introduction

Renal cell carcinoma (RCC) is a common malignant tumor of the urinary system and the second leading cause of urinary cancer-related death^1^. The onset of renal cancer is difficult to detect, as there are no typical clinical symptoms in the early stages or effective early diagnostic markers for renal cancer. In addition, renal cancer has already metastasized in approximately 30% of patients at the time of their initial diagnosis^2^. While the 5-year survival rate of early renal cancer can reach higher than 90%, it is significantly lower in advanced renal cancer, at approximately 10%. In the last decade, targeted drugs have given hope to patients with advanced renal cancer and improved the survival of affected patients. However, most patients treated with these drugs develop resistance within 6–15 months^3^. Therefore, studies aimed at exploring the potential mechanisms underlying of RCC development and metastasis are particularly important for supporting efforts to identify effective and reliable biomarkers and therapeutic targets that will improve the RCC survival rate.

The sequencing of the human genome revealed that over 80% of genes do not encode proteins, and the RNAs transcribed from these noncoding genes contain an important class of long noncoding RNAs (lncRNAs), which are more than 200 nucleotides in length. Recent research has shown that lncRNAs play important roles in tumor progression and metastasis. These noncoding RNAs can affect the transcription and translation of coding genes via multiple mechanisms, such as chromosome remodeling, transcriptional activation, or Inhibition, protein inhibition, and post-transcriptional modification.

In recent years, the use of high-throughput sequencing has led to the discovery of many lncRNAs associated with renal cancer^4–8^, such as the lncRNA HOTAIR, which is targeted and regulated by miR-141 in renal carcinoma cells^4^. Hirata et al.^5^ reported that MALAT1 promotes invasion in renal cancer by binding to EZH2, which is regulated by miR-205. Qiao et al.^6^ found that the overexpression of GAS5 inhibited RCC proliferation, invasion, and metastasis. Our research team also found that lncARSR levels are high in sunitinib-resistant RCC tissues. LncARSR promotes sunitinib resistance by competitively binding miR-34/miR-449 to facilitate AXL and c-MET expression in RCC cells^7^. Although some noncoding RNAs have been reported to be involved in the development and metastasis of renal cancer, the roles and mechanisms of these lncRNAs in renal cancer remain unclear.

Epidermal growth factor receptor (EGFR) is upregulated in many cancers including renal cancer^9–12^. EGFR often acts as an oncogenic driver in tumorigenesis. EGFR-AS1 is transcribed on the antisense strand of EGFR and has partial sequence complementarity with EGFR. Tan et al.^13^ reported that EGFR-AS1 mediated EGFR addiction and induced resistance to tyrosine kinase inhibitors (TKIs) in squamous cell carcinoma. The upregulation of EGFR-AS1 increased EGFR expression and predicted poor prognosis in hepatocellular carcinoma^14^. Furthermore, Hu et al.^15^ found that the knockdown of EGFR-AS1 decreased EGFR expression by reducing EGFR mRNA stability. However, the function and mechanisms of EGFR-AS1 in RCC have not been reported. The mechanism by which EGFR-AS1 regulates EGFR particularly requires further research.

In the present study, we found that EGFR-AS1 was expressed at high levels in RCC by screening and analyzing publicly transcriptome sequencing data from renal cancer (*n* = 703). Clinical data analyses suggested that high EGFR-AS1 expression predicted a poor prognosis in RCC patients. Subsequent mechanistic studies further identified that EGFR-AS1 promoted the expression of EGFR by enhancing its mRNA stability, thereby promoting the proliferation and metastasis of renal cancer cells. RNA pull-down and following mass spectrometry analysis identified proteins that could bind to EGFR-AS1, and HuR was validated to increase the stability of EGFR mRNA. These results indicated that further studies are necessary to elucidate the complex genetic rewiring driven by EGFR-AS1 in RCC.

## Materials and methods

### Microarray analysis

Microarray analysis for the expression of lncRNAs was performed to identify the probe sets uniquely mapped to lncRNAs, in which special way can evaluate the lncRNA expressions in RCC gene expression data^16^. The accession numbers for the microarray data are Gene Expression Omnibus database GEO: GSE40911, GSE61763, GSE76207, GSE82122, and TCGA datasets^17–20^. The differentially expressed genes with statistical significance were analyzed and identified using the R language. The threshold we used to screen upregulated or downregulated genes was a fold change >2.0 and a *P*-value <0.05.

### Patients and clinical samples

A total of 204 RCC tissues and paracancerous tissues were collected from patients who underwent RCC surgery at Changzheng Hospital (changzheng cohort), Second Military Medical University (Shanghai, China). These tissues were quickly frozen in liquid nitrogen immediately after surgery and then stored at −80 °C. All excised tissues were examined by pathologists. In this study, none of the patients received anti-cancer treatment before surgery. All samples were graded according to the 2010 AJCC tumor node metastasis (TNM) classification system and the criteria of the World Health Organization (WHO), and tumor grades were evaluated according to the WHO criteria. The median follow-up time for the 204 RCC patients was 68 months. All patients provided written informed consent. The Ethics Committee of the Changzheng Hospital of the Second Military Medical University approved the use of these organizations in this study.

### Cell lines and culture conditions

Renal cancer cell lines used in the experiment were all purchased from the American ATCC cell bank; 786O, OSRC-2, and KETR-3 were cultured in RPM11640 medium (Gibco) containing 10% fetal bovine serum (HyClone), and A498 and ACHN were cultured in MEM medium (Gibco) containing 10% fetal bovine serum. Renal cancer cell culture conditions were 37 °C, 5% CO_2_ saturated humidity incubator.

### Real-time polymerase chain reaction (RT-PCR)

Total RNA was isolated using TRIzol reagent (Invitrogen, USA) according to the manufacturer’s instructions. Real-time quantitative polymerase chain reaction (PCR) was performed on triplicate samples in a reaction mix of SYBR Green (Takara, China) by ABI 7900HT Fast Real-Time PCR System (Applied Biosystems, USA). The expression of indicated genes was normalized to endogenous reference control β-actin by using the 2^−△△Ct^ method. The primers were synthesized by Biosune (Sangon Biotech, China). Each quantitative reverse transcriptase PCR (qRT-PCR) reaction was performed in triplicate. Sequences of primers used for qRT-PCR in this study were shown in Supplementary Table S1.

### Cellular fractionation assay of RNA

Separation of nuclear and cytoplasmic RNA was performed using PARISTM kit (Ambion, AM1921) according to the manufacturer’s instructions. The β-actin mRNA was used as cytoplasmic control and U6 RNA as nuclear control. Cellular fractionation assay was verified in two RCC cell lines.

### 5′ and 3′ rapid amplification of cDNA ends

5′ and 3′ rapid amplification of cDNA ends (RACE) was performed to determine the transcriptional initiation and termination sites of lncRNA EGFR-AS1 using a SMARTer™ RACE cDNA Amplification Kit (Clontech, Palo Alto, CA) according to the manufacturer’s instructions. The gene-specific primers used for the PCR of the RACE analysis were 5′-GACGGGCAACGGCGTATTCTCAG-3′ (5′ RACE) and 5′- CCCACCTTGCCTTTGTCTCCTGTC-3′ (3′ RACE).

### RNA FISH

Fluorescence-conjugated EGFR-AS1 or EGFR mRNA probes were used for RNA FISH, which was performed as previously described^21^. Hybridization was performed using DNA probe sets (Ribobio, Guangzhou, China) according to the manufacturer’s instructions, and control cells were observed using an NA1.4 inverted Leica DMI6000 microscope (Leica, Heidelberg, Germany). The images were recorded using a Hamamatsu ORCA-R2 camera (Hamamatsu Photonics, Hamamatsu, Japan) and recorded with LAS AF software (Leica). RNA FISH experiments were performed in two RCC cell lines.

### Cell proliferation assay

Cell Counting Kit-8 (CCK-8; Dojindo Molecular Technologies, Inc., Kyushu, Japan) was used to assess cell proliferation ability, according to the manufacturer’s instructions. Cells were seeded into 96-well culture plates at a density of 2 × 10^3^ cells per well the day before transfection. The viability of RCC cells was assessed from five replicates in three independent experiments by CCK-8 after treated with indicated reagents at specific concentration for 48 h.

### Wound healing assay

RCC cells was seeded into six-well culture plates at a density of 5 × 10^5^ cells per well and cultured until the plates were confluent. The cell monolayers were scraped off in a straight line using a 10 μl pipette tip to create scratches, washed twice with phosphate-buffered saline, and the media replaced with serum-free media. Images were captured 0, 24 (18), and 48 (36) h after the initial scratches to assess cell migration.

### Transwell assay

The invasive capacity of RCC cells was evaluated based on the number of transfected cells that crossed Matrigel-coated Transwell inserts. Briefly, 3 × 10^5^ cells were seeded into 24-well plate-sized inserts (8-micron chamber; Corning Life Sciences, USA) using Matrigel (BD Biosciences, San Jose, USA). The cells were plated in serum-free medium, and the lower chamber contained medium plus 10% fetal bovine serum, which acted as a chemoattractant. After 24 h of incubation, cells that had not invaded the pores were carefully wiped off with cotton swab. All cells that had migrated from the upper part of the filter to the lower part were fixed with 4% paraformaldehyde and stained with 1% crystal violet. They were then counted and imaged (magnification ×100). These measurements were performed three times.

### Western blot analysis

Western blots were conducted using standard procedures. Cells were lysed to obtain proteins using RIPA. Proteins were separated by sodium dodecyl sulfate–polyacrylamide gel electrophoresi (SDS-PAGE) at the indicated concentration and transferred onto PVDF membranes. Antibodies were diluted to 1:1000 for EGFR (Abcam, Cambridge, UK), HuR (CST, Boston, USA), and β-actin (CST, Boston, USA). Secondary antibodies were then applied, including IRdye800-conjugated goat anti-rabbit IgG (Li-Cor Biosciences Inc., Lincoln, NE) and IRdye700-conjugated goat anti-mouse or anti-rabbit IgG (Li-Cor Biosciences Inc., Lincoln, NE) and detected using an Odyssey infrared scanner (Li-Cor Biosciences Inc., Lincoln, NE). Every western blots experiment was repeated three times.

### Cell transfection and lentivirus infection

Transfections were performed using a Lipofectamine 3000 kit (Invitrogen, Karlsruhe, Germany) according to the manufacturer’s instructions. Small interfering RNAs and their respective negative control RNAs (GenePharma) were introduced into cells at 75 pmol per well in six-well plates according to the manufacturer’s instructions, while 2.5 μg of plasmids were transfected per well. The cells were harvested 48 h after transfection. Sequences of the primers used for siRNAs and plasmid construction are shown in Supplementary Table S2.

The EGFR-AS1-overexpressing and control lentiviruses were purchased from Shanghai Heyuan Biotechnology and called lv-oeEGFR-AS1 and lv-NC, respectively. The CDS sequence containing EGFR-AS1 was amplified by PCR and cloned into the lentiviral vector pLV-CMV-X-PGK-EGFP-T2A-puro to construct the EGFR-AS1-overexpressing lentivirus. The appropriate amount of lentivirus was transfected into RCC cells, and the medium was changed after 48 h. After the cells were infected with lentivirus for 72 h, 1.5 µg/ml puromycin was selected for stable transformation screens. qRT-PCR and western blot analyses were used to verify the transfection efficiency of the lentiviruses. The EGFR-AS1 knockdown lentivirus was constructed for a small interference RNA and called lv-shEGFR-AS1.

### RNA pull-down assay and mass spectrometry

LncRNA EGFR-AS1 was transcribed in vitro from the vector pSPT19-lncRNA-EGFR-AS1 and biotinylated with biotinylated RNA labeling mix (Roche, Mannheim, Germany) and T7 RNA polymerase (Roche) and treated with RNase-free DNase I (Roche). It was also purified using RNeasy Mini Kit (Qiagen, Valencia, CA, USA). One milligram of whole-cell lysates from OS-RC-2 and 786-O cells were incubated with 3 μg of purified biotinylated transcripts at 25 °C for 1 h; streptavidin agarose beads (Invitrogen, Karlsruhe, Germany) were used to separate the complexes. RNA present in the pull-down material was purified using phenol:chloroform:isoamyl alcohol (125:24:1 pH = 4.3) and detected by RT-PCR analysis. At the same time, the relevant proteins were resolved by gel electrophoresis and visualized by silver staining. The binding proteins were also identified by mass spectrometry (H.Wayen Biotechnology, Shanghai).

### RNA immunoprecipitation

We performed RNA immunoprecipitation (RIP) experiments using a Magna RIP RNA-Binding Protein Immunoprecipitation Kit (Millipore, Bedford, MA, USA) according to the manufacturer’s instructions. We harvested and lysed 786-O and OS-RC-2 cells for RIP with HuR antibody. An aliquot of lysate was removed as an input control. RNA enrichment was determined by qRT-PCR and normalized to the input control.

### Statistical analysis

All statistical analyses were performed using SPSS Statistics software version 18 (SPSS Inc., USA). The data are presented as the mean ± SD or average grade. Depending on the type of data, the appropriate statistical methods were used, including the *t*-test, analysis of variance, chi-square test, and linear correlation analysis. The Kaplan–Meier method with the log-rank test was used to compare the survival rate of RCC patients based on dichotomized EGFR-AS1 expression. Survival data were evaluated using univariate and multivariate Cox proportional hazards models. Variables with a significant difference in the univariate analysis were assessed in the subsequent multivariate analysis based on Cox regression analyses. Two-sided *P*-values less than 0.05 indicated statistical significance.

## Results

### LncRNA EGFR-AS1 is upregulated in human RCC tissues

**Fig. 1:**
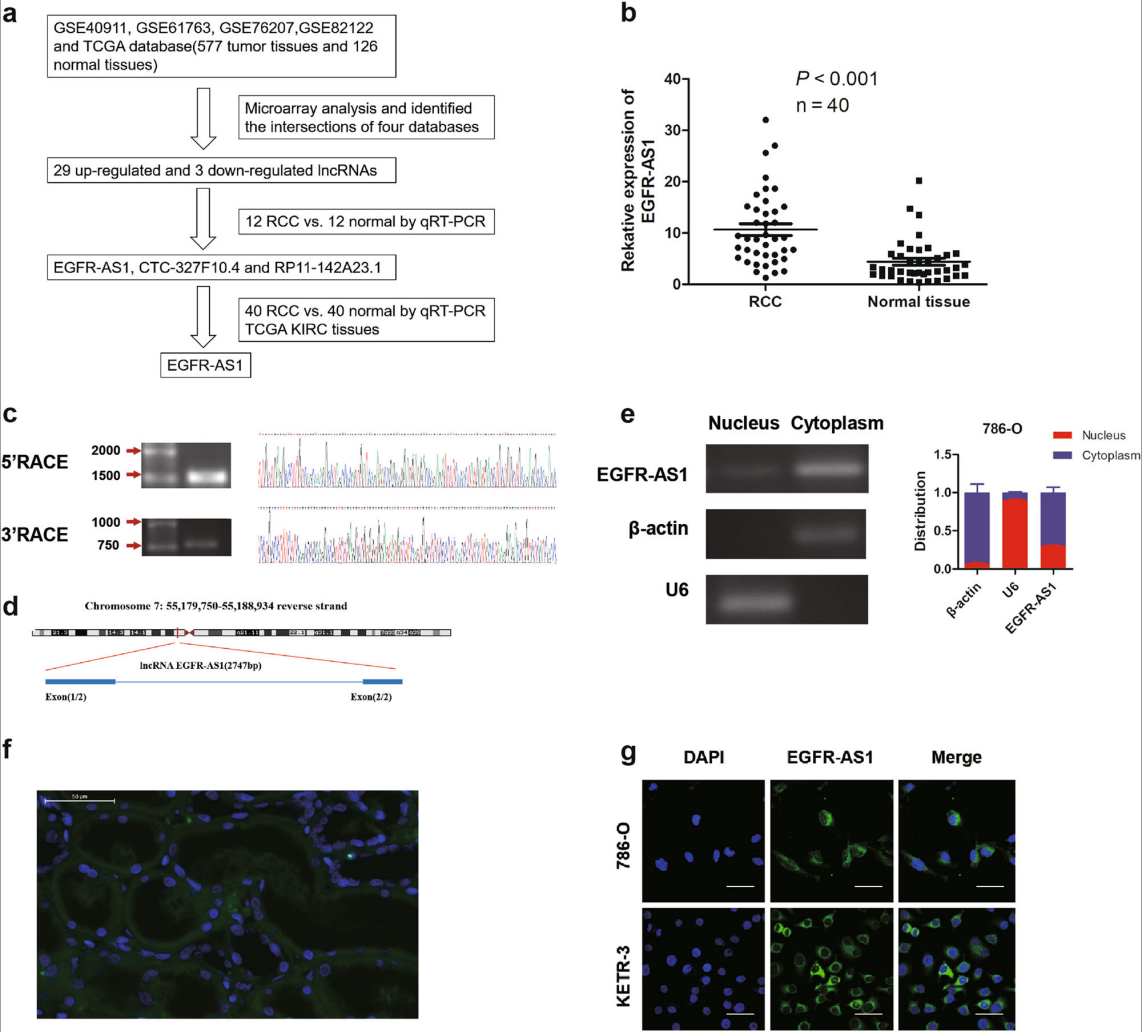
Identification of EGFR-AS1, which is upregulated in RCC tissues. **a** Flow chart of identification of EGFR-AS1 using GEO datasets and TCGA. **b** EGFR-AS1 expression between RCC samples and paired normal tissues was compared using qRT-PCR analysis (*n* = 40). *P* < 0.001 by the Mann–Whitney *U* test. **c** The full-length sequence of EGFR-AS1 was determined using 5′ and 3′ RACE assays. **d** Schematic annotation of the EGFR-AS1 genomic locus on chromosome 7: 55,179,750-55,188,934 reverse strand and composed of two exons in humans. Blue rectangles represent exons. **e** EGFR-AS1 identified in the subcellular fraction of 786-O cells using cellular fractionation assays. β-Actin and U6 are cytoplasmic and nuclear markers, respectively. **f** Representative images of RNA FISH analysis of EGFR-AS1 (green) in RCC tissues. Scale bar = 50 μm. **g** FISH analysis of EGFR-AS1 in 786-O and KETR-3 cells using a biotin-labeled RNA probe. Nuclei were stained with DAPI. Scale bar = 50 μm

First, we analyzed the differential lncRNA expression between RCC tissues and normal tissues in four GEO datasets (GSE40911, GSE61763, GSE76207, and GSE82122) and the TCGA database (577 tumor tissues and 126 normal tissues; Fig. 1a). From the intersection of the transcriptome sequencing data, 32 differentially expressed lncRNAs were initially obtained, including 29 upregulated and 3 downregulated lncRNAs (Supplementary Figure S1a). After qRT-PCR analysis of these lncRNAs in our own samples, we focused on three upregulated lncRNAs (EGFR-AS1, CTC-327F10.4, and RP11-142A23.1) (Fig. 1a). Next, we found that EGFR-AS1 was more strongly increased in RCC tissues than were CTC-327F10.4 and RP11-142A23.1 in a cohort including 40 pairs of RCC and normal tissues (Fig. 1b; Supplementary Figure S1b). Hence, we identified the lncRNA EGFR-AS1 as our research subject.

For further study, we performed RACE assay to identify the full sequence of EGFR-AS1 in 786-O cells according the sequence archived in the RefSeq database of NCBI (2747 bp; Fig. 1c; Supplementary Figure S1c). EGFR-AS1 is located on chromosome 7, near EGFR, and is composed of 2 exons (Fig. 1d). Then the coding potential of EGFR-AS1 was analyzed using Coding Potential Calculator (CPC) score, CPAT analysis, and PyhloCSF^22–24^, which all indicated that EGFR-AS1 does not encode a protein (Supplementary Figure S1d). The subcellular distribution assay suggested that EGFR-AS1 was mainly located in the cytoplasm of RCC cells and of cells in clinical RCC tissues (Fig. 1e–g).

### EGFR-AS1 facilitates the proliferation and invasion of renal cancer cells

**Fig. 2:**
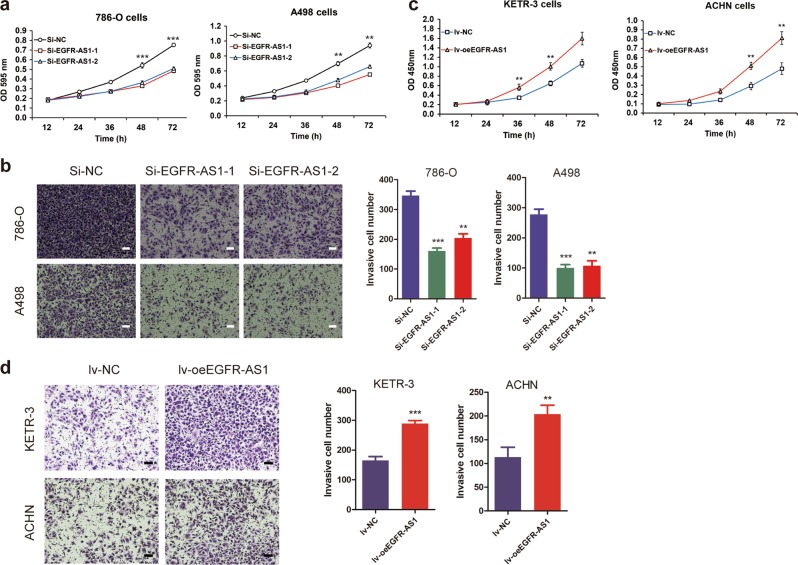
EGFR-AS1 knockdown suppresses RCC cell proliferation, migration, and invasion in vitro. **a** CCK-8 assay of EGFR-AS1 knockdown and control group RCC cells at the indicated times. **b** Left: Transwell assays were performed to evaluate cell invasion in EGFR-AS1 knockdown and control group RCC cells. Scale bar = 200 μm. Right: Statistical graph indicating the means ± SD of the number of cells in eight randomly selected high-power fields (magnification, ×200) counted from three independent experiments. **c** CCK-8 assay of EGFR-AS1 overexpression and control group RCC cells at the indicated times. **d** Left: Transwell assays were performed to evaluate cell invasion in EGFR-AS1 overexpressing and control group RCC cells. Scale bar = 200 μm. Right: Statistical graph indicating the means ± SD of the number of cells from eight random high-power fields (magnification, ×200) counted from three independent experiments. *P < 0.05, **P < 0.01, ***P < 0.001 by Student’s *t*-test

We transfected two small interference RNAs (siRNAs) against EGFR-AS1 into 786-O and A498 cell lines (Supplementary Figure S2a, b). Knocking down EGFR-AS1 significantly inhibited cell proliferation, as determined using cell proliferation assays (Fig. 2a). The wound healing assay showed that down-regulating EGFR-AS1 significantly inhibited cell migration (Supplementary Figure S2c). Similarly, transwell invasion assays revealed that EGFR-AS1 knockdown inhibited RCC cell invasion (Fig. 2b).

As the full-length sequence of EGFR-AS1 was obtained by 5′ and 3′ RACE experiments, EGFR-AS1 overexpression (lv-oeEGFR-AS1) and control lentivirus (lv-NC) were constructed and transfected into RCC cell lines (Supplementary Figure S2d). We found that the proliferative capacity of KETR-3 and ACHN cells was significantly increased upon EGFR-AS1 overexpression (Fig. 2c). The wound healing assay showed that EGFR-AS1 overexpression resulted in a faster KETR-3 cell migration rate than was observed in the control group (Supplementary Figure S2e). Additionally, EGFR-AS1 overexpression promoted RCC cell invasion, as determined using transwell invasion assays (Fig. 2d). Taken together, these results indicate that EGFR-AS1 promotes RCC cell proliferation and invasion.

### EGFR-AS1 knockdown suppresses tumor growth and metastasis in vivo

**Fig. 3:**
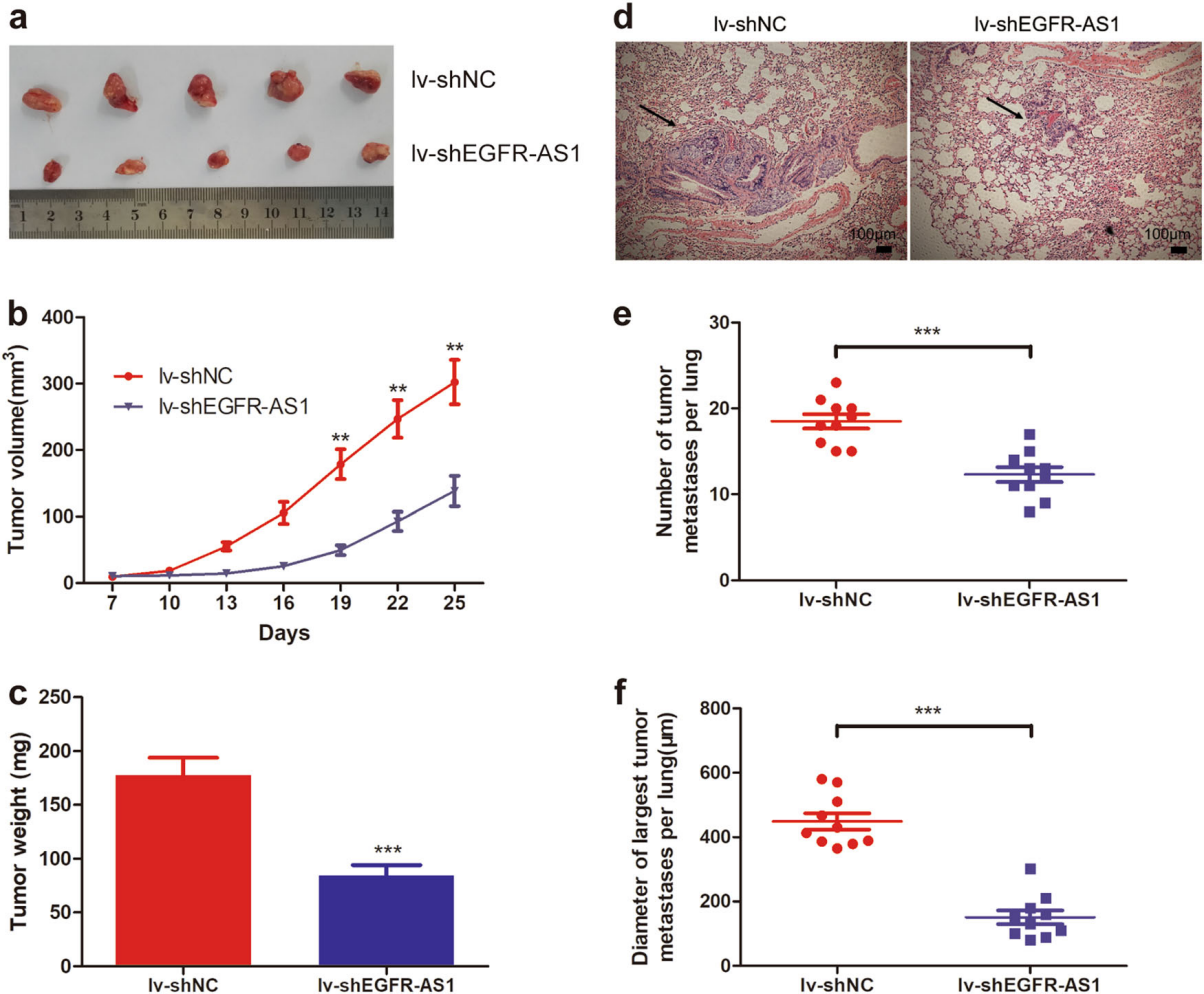
EGFR-AS1 knockdown suppresses RCC cell growth and metastasis in vivo. **a** Nude mice were given xenografts of EGFR-AS1 knockdown (lv-shEGFR-AS1) and control 786-O cells (5 × 10^6^ cells per site). The tumors were dissected and photographed after approximately 4 weeks (*n* = 5 per group). **b** The growth curve of EGFR-AS1 knockdown (lv-shEGFR-AS1) tumors compared to control 786-O tumors; bars indicate SD. **c** Tumor weights were measured after tumor removal. **d** Representative images of HE staining in metastatic nodules in the lungs of nude mice. The metastatic nodules are indicated by black arrows; scale bar = 100 μm. **e** The numbers of metastatic tumors in the lungs of nude mice were calculated and compared. **f** The diameter of the largest metastatic tumor in the nude mice lungs were calculated and compared. The results are presented as the mean ± SD for each group (n = 10). **P < 0.01, ***P < 0.001 by the Mann–Whitney *U* test

We constructed EGFR-AS1 interference lentivirus (lv-shEGFR-AS1) and control lentivirus (lv-shNC) according to the Si-EGFR-AS1-1 sequence (Supplementary Figure S3a). To determine the role of EGFR-AS1 in RCC growth in vivo, EGFR-AS1 knockdown or control 786-O cells were subcutaneously injected into nude mice. After several weeks of observation, we found that tumor volumes and weights were lower in the lv-shEGFR-AS1 group (Fig. 3a–c). To determine the effect of EGFR-AS1 on RCC metastasis in vivo, we established a lung metastasis mouse model, and the number and diameter of pulmonary metastasis lesions were smaller and fewer in the EGFR-AS1 knockdown group (Fig. 3d–f). These results suggest that EGFR-AS1 promotes RCC tumor growth and metastasis in vivo.

### EGFR-AS1 promotes RCC cell proliferation and invasion by upregulating EGFR expression

**Fig. 4:**
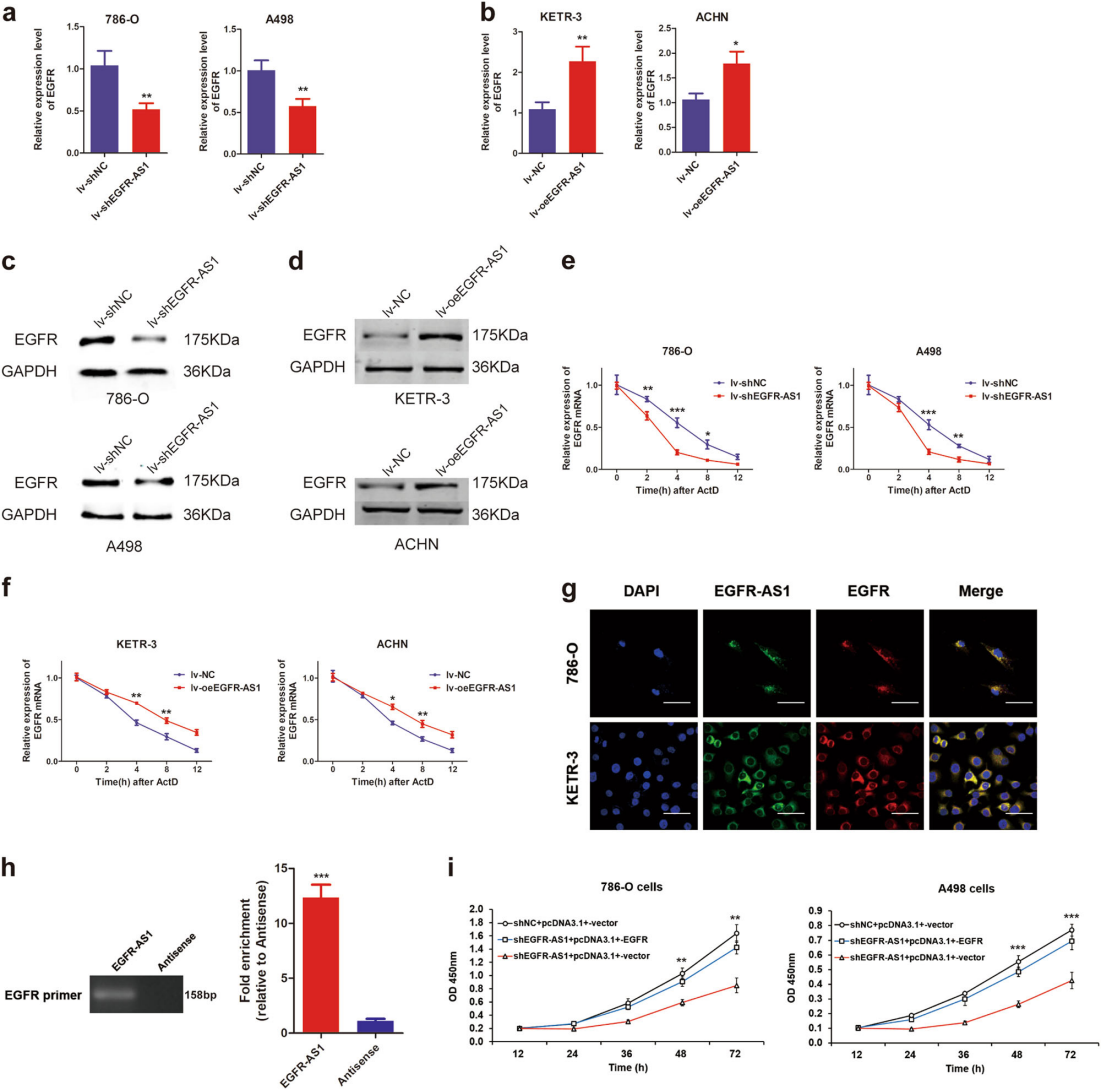
EGFR-AS1 promotes proliferation and migration in RCC cells by upregulating EGFR expression. **a** Relative expression of EGFR at the mRNA level between the lv-shNC and Lv-shEGFR-AS1 RCC cell lines. **b** Relative expression of EGFR at the mRNA level between the lv-NC and lv-oeEGFR-AS1 RCC cell lines. **c** Western blot analysis of EGFR protein expression between the EGFR-AS1 knockdown and control group. GAPDH was used as the internal control. **d** Western blot analysis of EGFR protein expression between the EGFR-AS1 overexpression and control group. **e, f** RNA stability assays were performed in RCC cell lines using Actinomycin D to disrupt RNA synthesis, and the degradation rate of the EGFR mRNA was measured and calculated over 12 h. EGFR mRNA levels were measured in the EGFR-AS1 knockdown (**e**) or overexpression (**f**) group and the NC group. **g** RNA FISH analysis of EGFR-AS1 (green) and EGFR mRNA (red) in 786-O and KETR-3 cells. The rightmost graph shows the colocalization of signals between the red signal (EGFR-AS1) and the green signal (EGFR). Pearson’s *R* = 0.696. Scale bar = 50 μm. **h** Agarose gel electrophoresis experiments of enriched product from the EGFR-AS1 RNA pull-down experiment in 786-O cells. EGFR-AS1 pull-down products were purified to obtain total conjugated RNA using RNA purification step in the RIP experiment. The antisense of EGFR-AS1 served as an internal control. **i** CCK-8 assay of EGFR-AS1 knockdown and control cells transfected with pcDNA3.1^+^-EGFR at the indicated times

Given the sequence complementarity of EGFR with EGFR-AS1, we first explored the relationship between their expression levels. qRT-PCR results showed that EGFR mRNA expression was decreased after EGFR-AS1 was knocked down in 786-O and A498 cells (Fig. 4a). Consistently, when EGFR-AS1 was overexpressed, EGFR expression was significantly increased (Fig. 4b). Moreover, western blot showed that EGFR protein expression was also reduced after EGFR-AS1 knockdown and was increased following EGFR-AS1 overexpression (Fig. 4c, d).

Due to the sequence complementarity of EGFR with EGFR-AS1 (Supplementary Figure S3b), and previous reports^15^, we speculated that EGFR-AS1 may affect EGFR expression by regulating EGFR mRNA stability in renal cancer. We found that EGFR-AS1 knockdown decreased EGFR mRNA levels after treatment with actinomycin D (ActD), a transcriptional inhibitor. This effect was most significant after 4 h, indicating that EGFR mRNA stability decreased after EGFR-AS1 was silenced (Fig. 4e). In line, EGFR-AS1 overexpression increased EGFR mRNA stability (Fig. 4f). The RNA FISH assay indicated that EGFR-AS1 colocalized with EGFR mRNA (Pearson’s *R* = 0.696) (Fig. 4g). Notably, EGFR-AS1 RNA pull-down products were purified to obtain total conjugated RNA and PCR assays showed that EGFR mRNA was detected in the final products (Fig. 4h), indicating that EGFR-AS1 specifically bound to EGFR mRNA. Additionally, overexpressing EGFR rescued the reduction in cell proliferation capacity caused by EGFR-AS1 knockdown (Fig. 4i). These results indicate that EGFR-AS1 binds to EGFR mRNA and increases its stability in RCC cells.

### EGFR-AS1 maintains EGFR mRNA stability by binding to HuR

**Fig. 5:**
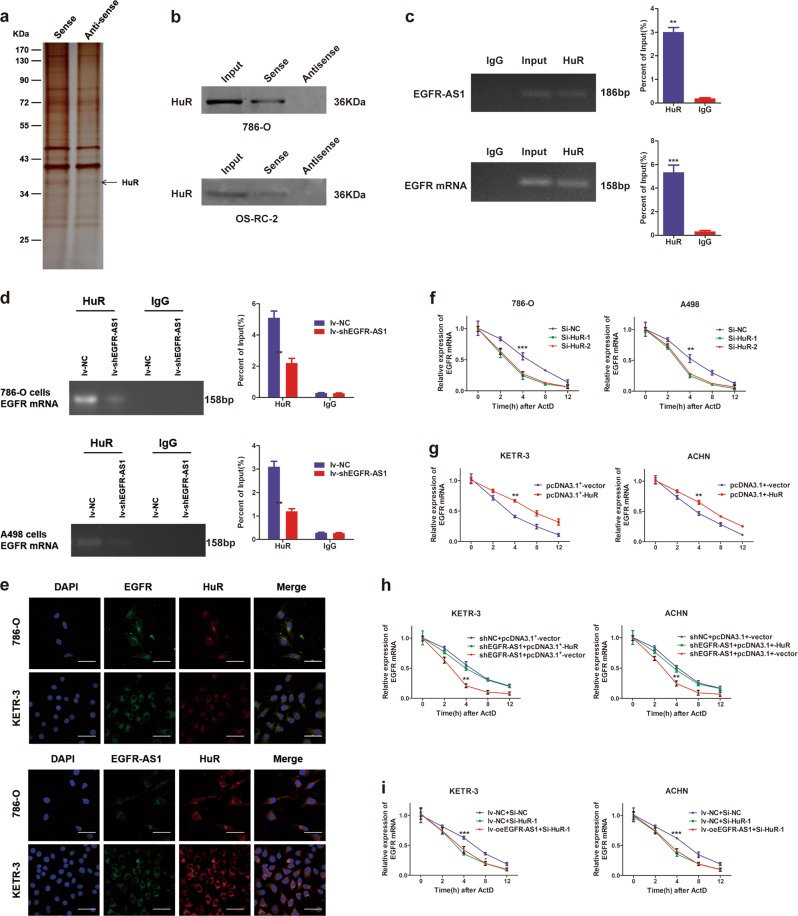
EGFR-AS1 promotes the maintenance of EGFR mRNA stability by binding to HuR. **a** Silver staining SDS-PAGE gel of electrophoretically separated proteins immunoprecipitated with EGFR-AS1 and its antisense RNA in 786-O cells. **b** RNA pull-down assay was performed in 786-O and A498 cells using biotinylated EGFR-AS1 or antisense RNA probe transcribed in vitro and detected by western blots. **c** Upper: RIP assays were performed in 786-O cells using HuR antibody to detect EGFR-AS1 RNA enrichment in immunoprecipitated complexes. IgG is the negative control. Lower: RIP assays were performed in 786-O cells using HuR antibody to detect EGFR RNA enrichment in immunoprecipitated complexes. **d** RIP assay of the enrichment of EGFR mRNA with HuR between the EGFR-AS1 knockdown and NC group in RCC cells. IgG was used as an internal control. **e** Upper: RNA FISH analysis of EGFR mRNA (green) and immunofluorescence detection of HuR (red) in RCC cells. The rightmost graph shows colocalization between the green signal (EGFR) and the red signal (HuR). Pearson’s *R* = 0.583. Scale bar = 50 μm. Lower: RNA FISH analysis of EGFR-AS1 (green) and immunofluorescence detection of HuR (red) in RCC cells. Pearson’s *R* = 0.416. **f** The rate of degradation of the EGFR mRNA between the HuR knockdown and control group using RNA stability assays in RCC cells. **g** The rate of degradation of the EGFR mRNA between the HuR overexpressing and control group using RNA stability assays in RCC cells. **h** The rate of degradation of the EGFR mRNA in the EGFR-AS1 knockdown and control cells transfected with pcDNA3.1^+^-HuR over 12 h in KETR-3 and ACHN cells. **i** The rate of degradation of the EGFR mRNA in the EGFR-AS1 overexpressing and control cells transfected with HuR siRNA over 12 h

Recently, a number of studies have reported that certain lncRNAs are involved in the regulation of signaling pathways by interacting with specific proteins^25,26^. We next performed RNA pull-down, followed by SDS-PAGE and mass spectrometry assays to identify proteins associated with EGFR-AS1 (Fig. 5a). We identified 634 proteins in the sense group and 631 proteins in the antisense group (data not shown). After comparing different proteins between the sense and antisense groups, HuR was identified as potential binding candidate for EGFR-AS1 (Supplementary Table S3), which was validated by RNA pull-down and RIP assays (Fig. 5b, c). We also found a direct interaction between HuR and EGFR mRNA by RIP assay (Fig. 5c), which indicated a close relationship among HuR, EGFR-AS1, and EGFR mRNA.

HuR, also known as ELAVL1, is an important RNA-binding protein that regulates mRNA stability^27^. It binds to AU-rich elements (AREs) in the mRNAs of certain inflammatory factors (such as VEGF, COX-2, IL-8, and IL-6), and enhances their mRNA stability. EGFR mRNA also includes some AREs, which indicate the binding potential of EGFR with HuR. In RCC cells, RIP assays showed that EGFR-AS1 knockdown reduced the ability of HuR to bind EGFR mRNA (Fig. 5d). RNA FISH and immunofluorescence experiments indicated that EGFR and EGFR-AS1 colocalized with HuR (Pearson’s *R* = 0.416 and 0.486, respectively) (Fig. 5e). These results further verify the close regulatory relationship among EGFR-AS1, EGFR, and HuR.

Finally, we explored the effect of HuR on EGFR. HuR siRNAs were designed and verified for their interference efficiency (Supplementary Figure S3c). HuR knockdown decreased EGFR expression in RCC cells (Supplementary Figure S3d). Moreover, silencing HuR reduced the stability of EGFR mRNA (Fig. 5f). Consistently, HuR overexpression increased EGFR mRNA levels and promoted its stability (Fig. 5g; Supplementary Figure S3e, f). Additionally, HuR overexpression rescued the reduction in EGFR mRNA stability caused by EGFR-AS1 knockdown (Fig. 5h). HuR knockdown eliminated the effect of EGFR-AS1 overexpression on EGFR mRNA in RCC cells (Fig. 5i). Based on these results, we conclude that EGFR-AS1 maintains the stability of EGFR mRNA by binding to HuR, thereby promoting RCC cell proliferation and metastasis.

### EGFR-AS1 upregulation is associated with RCC progression and poor prognosis

**Fig. 6:**
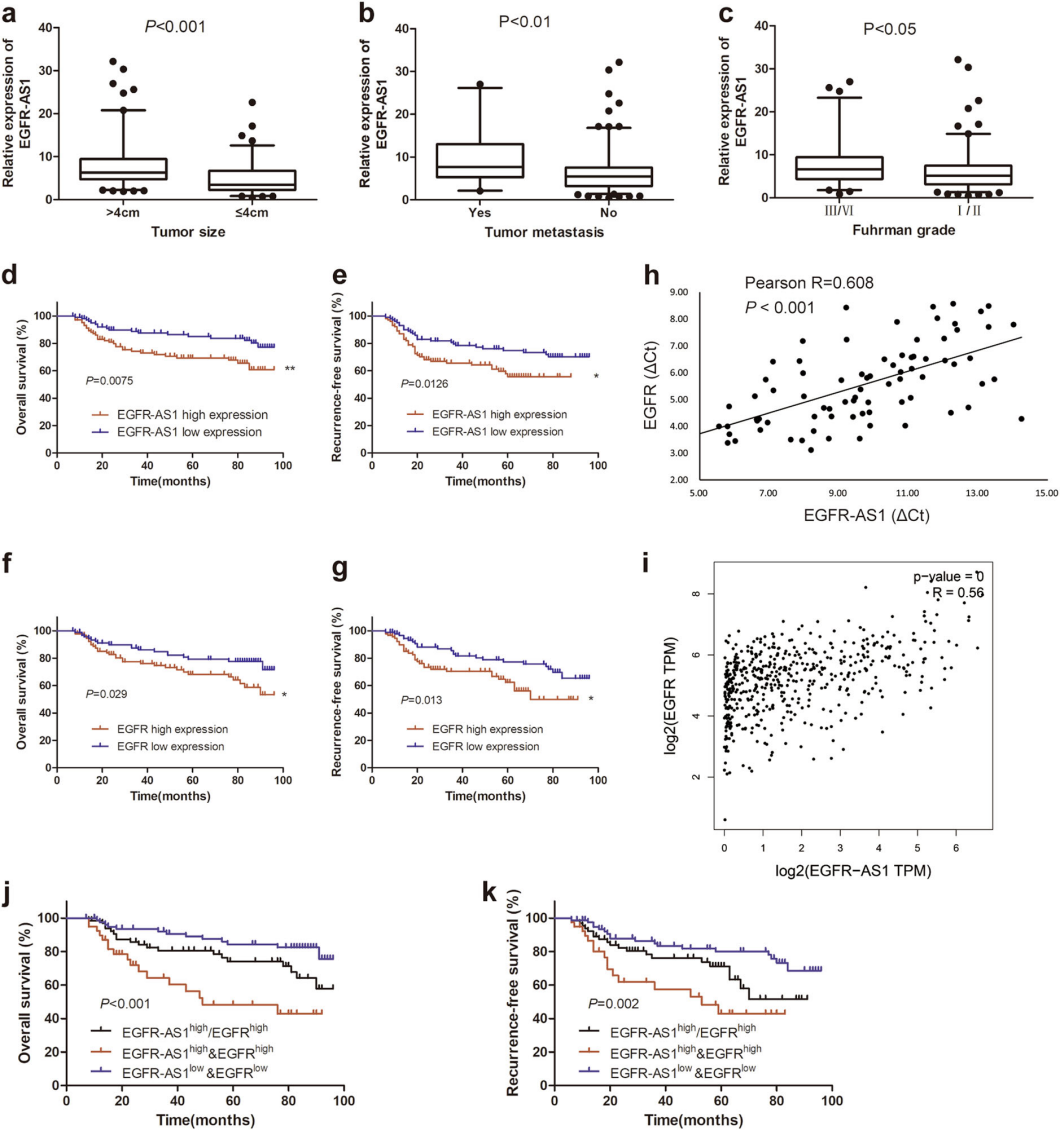
Combining EGFR-AS1 and EGFR exhibits improved prognostic value. **a** EGFR-AS1 expression between tumors >4 cm (*n* = 119) and tumors ≤4 cm (*n* = 85) in RCC samples analyzed using qRT-PCR. *P* *<* 0.001 by the Mann–Whitney *U* test. **b** EGFR-AS1 expression between RCC samples with tumor metastasis (*n* = 31) and without tumor metastasis (*n* = 173) analyzed using qRT-PCR. *P* < 0.01 by the Mann–Whitney *U* test. **c** EGFR-AS1 expression between Fuhrman III/IV grade (*n* = 52) and Fuhrman I/II grade (*n* = 152) RCC samples analyzed using qRT-PCR. *P* < 0.05 by the Mann–Whitney *U* test. **d**, **e** Kaplan–Meier analysis of the overall survival (**d**, *P* = 0.0075) or recurrence-free survival rate (**e**, *P* = 0.0126) of RCC patients with high or low EGFR-AS1 expression. **f**, **g** Kaplan–Meier analysis of the overall survival (**f**, *P* = 0.029) or recurrence-free survival rate (**g**, *P* = 0.013) of RCC patients with high or low EGFR expression. **h**, **i** The Pearson correlation analysis of the transcription level of EGFR-AS1 and EGFR in our data (**h**, *n* = 80, *P* = 0.038) and TCGA database(**i**, *P* = 0.019). **j**, **k** Kaplan–Meier analysis of the overall survival (EGFR-AS1^high^&EGFR^high^ vs EGFR-AS1^high^/EGFR^high^ and EGFR-AS1^low^&EGFR^low^, *P* < 0.001) or recurrence-free survival rate(*P* = 0.002) of RCC patients with high or low EGFR-AS1 and high or low EGFR

We analyzed the correlation between EGFR-AS1 expression and clinicopathological traits in 204 cases of RCC patients (Changzheng cohort). The results suggested that EGFR-AS1 was expressed at substantially higher levels in tumors >4 cm than in tumors ≤4 cm (*P* < 0.001), in Fuhrman III/IV grade tumors than in Fuhrman I/II grade tumors (*P* < 0.05), and in the distant metastasis group than in the no metastasis group (*P* < 0.01) (Fig. 6a–c). These data indicated that EGFR-AS1 was involved in RCC progression.

Based on EGFR-AS1 expression by qRT-PCR, 204 RCC patients were divided into a high EGFR-AS1 group (*n* = 102) and a low EGFR-AS1 group (*n* = 102). High EGFR-AS1 expression was related to larger tumor size (*P* = 0.007), higher Fuhrman grade (*P* = 0.025), advanced TNM stage (*P* = 0.023) and distant metastasis (*P* = 0.032) (Table 1). The survival analysis suggested that the overall survival and recurrence-free survival rates were significantly better in the low EGFR-AS1 group than in the high EGFR-AS1 group (Fig. 6d, e; Supplementary Figure S4a). In addition, a multivariate analysis identified EGFR-AS1 expression as an independent prognostic factor in RCC patients (Table 2). Then, 182 RCC patients in Changzheng cohort were divided into a high EGFR group (*n* = 91) and a low EGFR group (*n* = 91) according to EGFR RNA expression. The survival analysis indicated that the low EGFR group had better overall survival and recurrence-free survival rates than the high EGFR group (Fig. 6f, g; Supplementary Figure S4b). EGFR-AS1 levels were positively correlated with EGFR mRNA levels in 80 RCC tumor specimens, which was validated in the TCGA databases (Fig. 6h, i). Although either high EGFR-AS1 or EGFR in RCC predicted a poor prognosis (Fig. 6d–g), RCC patients with both elevated EGFR-AS1 and EGFR expression displayed an even worse prognosis(Fig. 6j, k), indicating the superior prognostic value of combining the two parameters vs. using EGFR-AS1 or EGFR alone. These results indicated that EGFR-AS1 represents a new prognostic factor for RCC patients.

**Table 1:** Correlations between EGFR-AS1 expression and clinicopathological features

Variables	Low EGFR-AS1 (*n* = 102)	High EGFR-AS1 (*n* = 102)	*P* value
**Gender**			0.765^*^
Male	70	68	
Female	32	34	
**Age**			0.884
≤60	65	66	
å 60	37	36	
**Tumor size, cm**			0.007^*^
≤4 cm	52	33	
å 4 cm	50	69	
**Fuhrman grade**			0.025^*^
I/II	83	69	
III/VI	19	33	
**TNM stage**			0.023^*^
I/II	78	63	
III/VI	24	39	
**Distant metastasis**			0.032^*^
No	92	81	
Yes	10	21	

^*^P values <0.05 were considered statistically significant

**Table 2:** Univariate and multivariate analyses of factors associated with overall survival in RCC patients

Variable	Univariate	Multivariate		
		HR	95% CI	*P* value
**EGFR-AS1 expression**				
High vs Low	0.007	2.204	1.145–4.241	0.018^*^
**Tumor size**				
>4 cm vs ≤ 4 cm	0.001	1.69	0.843–3.114	0.112
**Fuhrman grade**				
Yes vs No	0.004	0.752	0.325–1.651	0.385
**Metastasis**				
Yes vs No	0.001	5.302	1.528–20.734	0.021^*^

^*^P values <0.05 were considered statistically significant

*HR* Hazard ratio, *CI* Confidence interval

## Discussion

In recent years, newly discovered lncRNAs have emerged as important players in the development of numerous human diseases, especially cancer. Researchers often use single-center tissue sequencing data to identify new valuable lncRNAs. In the present study, utilizing publicly available transcriptome sequencing data from renal cancer (*n* = 703) and integrating bioinformatics analyses, we screened and identified a valuable lncRNA, EGFR-AS1. The large sample sequencing data help reduce the heterogeneity of different groups and increase the reliability of the results. EGFR-AS1 was upregulated in renal cancer tissues and high EGFR-AS1 expression in RCC patients was positively correlated with advanced TNM stage. Moreover, high EGFR-AS1 expression predicted a poor prognosis of RCC patients, and it may serve as an independent prognostic indicator. These results suggested that EGFR-AS1 may play a role in RCC progression.

EGFR-AS1, which is located at chromosome 7p11.2, is an antisense transcript of EGFR. EGFR-AS1 shares a complementary sequence with EGFR, which lays the foundation for its regulation of EGFR. EGFR is known to play a key role in the progression of various cancers, including renal cancer. In pulmonary cancer, gefitinib, a selective inhibitor of EGFR, has been applied in clinical treatment and has been shown to improve patient survival. The high EGFR expression observed in renal cancer tissues was closely related to the development and metastasis of renal cancer^12,28^. The elevated EGFR levels could lead to the activation of several downstream signaling pathways, including the MAPK, PLCγ, STAT, and PI3K/AKT pathways, in cancer cells. Abouzid and co-workers^29^ designed and synthesized selective EGFR-TK inhibitors, 4,6-quinazolinediamines, which effectively inhibited RCC cell proliferation. A Phase I clinical study found that the combination treatment of EGFR and VEGFR inhibitors was generally well tolerated and showed encouraging antitumor activity in patients with advanced renal cancer^30^. However, further studies are necessary to assess the effectiveness and safety of EGFR inhibitors in RCC treatment. In our findings, the upstream regulatory mechanism of EGFR may provide a new perspective for the synergistic inhibition of EGFR.

EGFR-AS1 has been reported to play oncogenic roles in hepatocellular carcinoma and gastric cancer^14,15^. In our study, we found that the inhibition of EGFR-AS1 repressed RCC cell proliferation and migration in vitro and in vivo. Tan et al.^13^ found that targeting EGFR-AS1 with a Locked Nucleic Acid (LNA) in vivo was sufficient to induce sustained squamous cell carcinoma regression in comparison to controls. EGFR-AS1 could potentially be targeted through RNA interference (RNAi)-based strategies that have entered clinical testing^31^. Based on previous research and the close relationship of EGFR-AS1 and EGFR^14,15^, we identified that EGFR-AS1 directly bound to EGFR mRNA and inhibited its degradation in renal cancer. Modulating mRNA stability is a more effective strategy than producing proteins de novo for cancer cells to allow rapid adaptation and maximum cell survival^32^. RNA pull-down and mass spectrometry analysis revealed that HuR interacted with EGFR-AS1, and knocking down HuR eliminated the effect of EGFR-AS1 on EGFR mRNA in RCC cells. This finding indicates that EGFR-AS1 requires HuR to maintain EGFR mRNA stability. HuR is involved in regulating the stability of mRNAs, such as VEGF mRNA^33^, by binding to AREs in the mRNA 3′ UTR^27,34^. EGFR mRNA also contains some AREs, which indicates the possibility of EGFR binding HuR. Our study demonstrated that EGFR-AS1 maintains the stability of EGFR mRNA by binding to HuR, thereby promoting proliferation and metastasis of RCC cells.

In human cells, there are four known EGFR isoforms (A–D), of which isoform A, C, and D are known to be translated^35,36^. EGFR isoform B, C, and D are soluble EGFR isoforms that lack the intracellular domain^37^. Our research evaluated EGFR expression as a single entity. However, it is notable that soluble EGFR isoforms, especially isoform D, may affect the responsiveness of cancer cells to the EGFR inhibitor^38^. Tan et al.^13^ found that the knockdown of EGFR-AS1 was sufficient to increase the isoform D:isoform A ratio, especially in G/G genotype squamous cell cancer cells, with consequent increased sensitivity to TKIs. However, we did not find that EGFR-AS1 regulated different isoforms of EGFR in RCC cell lines (Supplementary Figure S4c, d). The results were confirmed by the TCGA analyses (Supplementary Table S4). This could be due to tumor heterogeneity of different tumors. Even so, further studies are necessary to confirm the relationship between EGFR-AS1 and the different EGFR isoforms. It is worth noting that EGFR-AS1 is predominantly localized in the cytoplasm. Whether EGFR-AS1 participates in EGFR mRNA translocation between the nucleus and cytoplasm and whether EGFR-AS1 is involved in a coordinated transport mechanism require further study. In addition, there are limitations related to the limited sample number and the upstream mechanisms of EGFR-AS1 expression in renal cancer. In the future, we will study the regulatory mechanisms of EGFR-AS1 upregulation in renal cancer.

In conclusion, our study demonstrates that EGFR-AS1 predicts a poor prognosis of RCC patients. EGFR-AS1 enhances the malignant phenotype of RCC cells by enhancing HuR-mediated mRNA stability of EGFR. Our data also provide biological rationales for EGFR-AS1 as a prognostic biomarker and potential therapeutic target for RCC.

## Supplementary information

Supplementary Table

Supplement Figure

Supplementary information
