# Supplementary material for: Long noncoding RNA EGFR-AS1 promotes cell growth and metastasis via affecting HuR mediated mRNA stability of EGFR in renal cancer
Source: Cell Death Dis. 2019 Feb 15;10(3):154. doi: 10.1038/s41419-019-1331-9 (PMC6377662; doi:10.1038/s41419-019-1331-9)
Supplement: Supplementary file 1 — Supplementary Table [file 41419_2019_1331_MOESM1_ESM.docx]

**Supplementary Table**

| **Table S1. Sequences of primers used for qRT-PCR in this study** | |
| --- | --- |
|  |  |
| EGFR-AS1 Forward | CCATCACGTAGGCTTCCTGG |
| EGFR-AS1 Reverse | GCATTCATGCGTCTTCACCTG |
| CTC-327F10.4 Forward | AGGCTATTGAGGCAGATT |
| CTC-327F10.4 Reverse | GGTAGTTGCCATTTAGTTGTG |
| RP11-142A23.1 Forward | GGGTTGTCACCGTGTTCC |
| RP11-142A23.1 Reverse | CTGCCTTCAGAGTGGTTGG |
| EGFR Forward | TGGTCAAGTGCTGGATGATAGA |
| EGFR Reverse | ACGGTAGAAGTTGGAGTCTGTA |
| β-actin Forward | AATCGTGCGTGACATTAAGGAG |
| β-actin Reverse | ACTGTGTTGGCGTACAGGTCTT |
| U6 Primer S | GCTTCGGCAGCACATATACTAAAAT |
| U6 Primer A | CGCTTCACGAATTTGCGTGTCAT |
| HuR Forward | AGCCTGTTCAGCAGCATTGGTG |
| HuR Reverse | AGCCGTTCAGCGTGTTGATCG |
| GAPDH Forward | AGAAGGCTGGGGCTCATTTG |
| GAPDH Reverse | AGGGGCCATCCACAGTCTTC |
| EGFR IsoA Forward | ACTCTGAGTGCATACAGTGC |
| EGFR IsoA Reverse | TCGTTGGACAGCCTTCAAGAC |
| EGFR IsoD Forward | ACTCTGAGTGCATACAGTGC |
| EGFR IsoD Reverse | TGAAGGCATGAGGCTCAGTG |
| EGFR IsoC Forward | TTGCCGCAAAGTGTGTAACG |
| EGFR IsoC Reverse | AGGGAACAGGAAATATGTCGAA |

| **Table S2. Sequences of primers used for siRNAs and plasmid construction in this study** | |
| --- | --- |
|  |  |
| EGFR-AS1-1 sense | CUGCCUGCAAAUCCUUUAATT |
| EGFR-AS1-1 antisense | UUAAAGGAUUUGCAGGCAGTT |
| EGFR-AS1-2 sense | GAGGAGCACAUUGGAUAAATT |
| EGFR-AS1-2 antisense | UUUAUCCAAUGUGCUCCUCTT |
| HUR-homo-1 sense | CAGCAUUGGUGAAGUUGAATT |
| HUR-homo-1 antisense | UUCAACUUCACCAAUGCUGTT |
| HUR-homo-2 sense | CCAGUUUCAAUGGUCAUAATT |
| HUR-homo-2 antisense | UUAUGACCAUUGAAACUGGTT |
| pcDNA3.1+-HuR sense | GACACAAGCTTGCCACCATGTCTAATGGTTATGAAGAC |
| pcDNA3.1+-HuR antisense | GACACGAATTCTTATTTGTGGGACTTGTTGGTTTTGAAGG |

| **Table S3. Mass spectrometry analysis of the proteins pulled down by EGFR-AS1 in 786-O cells** | | | | |
| --- | --- | --- | --- | --- |
| Hits | Protein description | Peptides | Coverage (%) | Avg. Mass(Da) |
| 1 | Hornerin OS | 6 | 3 | 282389 |
| 2 | ELAV-like protein 1 | 6 | 10 | 36092 |
| 3 | Heterogeneous nuclear ribonucleoprotein H | 6 | 9 | 49229 |
| 4 | Caveolin-1 | 4 | 20 | 20472 |
| 5 | Zinc finger CCCH-type antiviral protein 1 | 4 | 6 | 101431 |
| 6 | Nuclear export mediator factor NEMF | 3 | 11 | 49859 |
| 7 | Heterogeneous nuclear ribonucleoprotein F | 2 | 3 | 45672 |
| 8 | Death-inducer obliterator 1 | 2 | 1 | 243870 |

| **Table S4. The correlation analysis of EGFR-AS1 with EGFR isoforms in TCGA KIRC database** | | |
| --- | --- | --- |
| **genes** | **Pearson r** | ***P*-value** |
| EGFR-AS1 vs EGFR Isoform A | 0.225 | 1.53E-07 |
| EGFR-AS1 vs EGFR Isoform D | 0.203 | 2.38E-06 |
| EGFR-AS1 vs EGFR Isoform C | 0.339 | 7.95E-16 |
| EGFR-AS1 vs EGFR Isoform D/A | -0.021 | 0.635 |

Analytical method: upper quartile normalized RSEM count estimates (75%)
