# Supplementary material for: Long noncoding RNA EGFR-AS1 promotes cell growth and metastasis via affecting HuR mediated mRNA stability of EGFR in renal cancer
Source: Cell Death Dis. 2019 Feb 15;10(3):154. doi: 10.1038/s41419-019-1331-9 (PMC6377662; doi:10.1038/s41419-019-1331-9)
Supplement: Supplementary file 2 — Supplement Figure [file 41419_2019_1331_MOESM2_ESM.pdf]

Figure S1

a

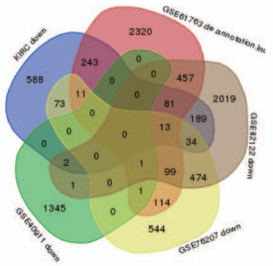

b

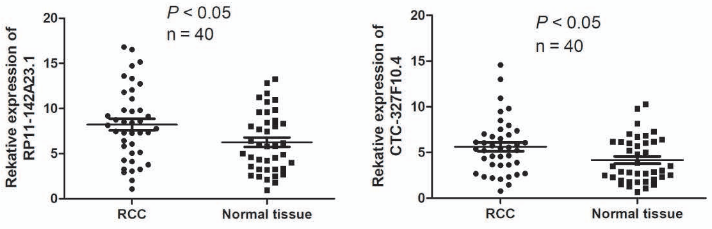

c

5'-3'

CATATTCTGGAATTCAGGTCACCTTTTACATCTGCCTGGAGTTGGGTGAGCGCCTCACGAATGACTCGGCAGGACTGACTGC
CACTGCTAACCAGGCGGATGCAAGCAATGAGGAGGACCTGCCCAGGCCGGGTGGGTGTCTGCTCCCTCCCTCTGGTCCCTG
GCACATCTCTGGACCCCCTGCCCTGCTGTCGGAGAGAGATGACGGGCAACGGCGTATTCTCAGAGACAGGGCCTGCCTGCA
AATCCTTTAAAGTCAATGGGATGAAATGTATACCCATTGTTAGAAAAAAATAGGACTTAGCAAGTTGAGTGCAAATAACTGATG
CAAGACTGGGATGGAGATGGGAGGGGTTTGGGGCAAAAGCAGAAGTCTTTCTGGGTCCGCACCAGCTGTGAAATACCTGGC
TTGTTGTTCTGTGCCTGTCTCCAGCACCCAGGCAGGGCTACCTGACCACTCTGCTCTCTCAGCCCCGCCCTGGCTCTGGAGC
GAGCCTGTGGAAAGGGGGACACTTAGCCAAGGCCCCAGCCACATAGCAGCAGCAGCTGCGCCCTCTGTCAGCTCCTTGCAC
CTCCTCACTGGGCCTCCTGCAAGGCACCTGCTCCACTCCACCACTATCACCTGGGTCCTCCTGGCCTTGGCCTGGCTTGCTT
ACCTTGTTATCAAGTCCTGAATGGGGGAAGCAATATTCCTTCTCCACTACAAATCACCACAGTATTCACAAAGAATTCCAGAGA
AATAAGAACAGAGACATCAGACCACACTGAGCACTCAATAAAGAGAAAATTCTTCAAAGGTAGCTGATTGATGAGAGTTTCCA
CATGCAGATGGGACAGGCACTGATTTGTGCACAAGAAGATCAACTTGATTGAATCCAAAATAAAGGAATGTGTGTGTGCTGCA
TGCACGCACACACATATCCCCATGGCAAACTCTTGCTATCCCAGGAGCGCAGACCGCATGTGAGGATCCTGGCTCCTTATCT
CCCCTCCCCGTATCTCCCTTCCCTGATTACCTTTGCGATCTGCACACACCAGTTGAGCAGGTACTGGGAGCCAATATTGTCTT
TGTGTTCCCGGACATAGTCCAGGAGGCAGCCGAAGGGCATGAGCTGCGTGATGAGCTGCACGGTGGAGGTGAGGCAGATG
CCCAGCAGGCGGCACACGTGGGGGTTGTCCACGCTGGCCATCACGTAGGCTTCCTGGAGGGAGGGAGAGGCACGTCAGTG
TGGCTTCGCATGGTGGCCAGAAGGAGGGGCACATGGACCCCTTCCAGGTGAAGACGCATGAATGCGATCTTGAGTTTCAAAA
TACGTACTCATGGAGGAAAAGCTGTGCCTGCAAAAGACCTAGCACAGGGACGTTTACGCAGGGCTGTGAAGTGACAGATGCA
GTGGGAGAGGGCCCCTCCTGGGTGCATCTGGGGATTCCCCATGACAGAGAGGCCCAGGCAACAGTGGCCATGAGGAGCAC
ATTGGATAAAGGAGGAGTCGGAGTCACCTGATCTCTGAGTTTGGGAACTGATAGTATCTTTGTTATGAAGACCTCCGCCTCAA
GGTTGAGGATGCTGTGTTTTAAAATATCATGAGGGCCTGTAGGAATCTGTGTGGGGTCCGGAACACCCTGGGTAATGACTGA
CCCTGCACATCAGGAACGCTGGCTGTGGGTGCTGCAGAGGACAAGCGATGGAGAAGGCATCCGGGGAGCACCGAGCCAGC
AGGGAGAAAGGCCTCCCTTCCCCACAGGCCAGGCTTGGCCCTGACTGTGCTCTGGGAAATGGGTGGGCATTTGGGCTGGG
GACCCTGCCCACAGCACCTCTGCAAAGAGTAGCTGGATAAGCTCTTTCAATAGACCAGTCCCAGGTTTTGAAATGGACAGAG
CATTCAATCTACAGTGACTAAAGGCTGCCTGGCTGCCCGGGACCCATTTCTAAAGAGAAGTGGTCTCTCTGTGCTGTGCCCC
CAGGCTCCCTATGGGAAATCCATGCTGCACTGAGTCAGGCATCTGCTGCCCTGCTAATTCCGGCTGGCTGCCAAGGCAGGG
GCCTTCCTTTGACAGAGCCATAAATACAGACTTTATTTTAACCCTTCTGCTATTCTTGGGCTGAGGAAGCTAAATTTATTTGCAA
TCAGGCACACAATGGGGCCCTCTTTTCTGTCTGACTGAGAATGAGGGAATCCCCAATTTCCACCCATAAATTCTCTTTCTCTTT
AAAATACAAATGGTGGTGACCTTTTATTCAGATATGGAAAAGAACACACAGACTGTAGCAGAAAGCATCCACAGCTGCTTTTCA
CATCTCAGCAATGCCTATGTTTTGAGTGTGGACTTGGGCAAGTTAGTTTCTCTGCAGAAGTGAACACACTGAGCCAGGCTCTG
AGATAGGGTGCTGCTCCAGGGTGCCCGGGCAGGTCAGGAGCAACAGGCTGGCGGGAGGCAGGGTGGAGATAGGAGACAG
GAGACAAAGGCAAGGTGGGGCGAGGGGACACAGACAGTGGACCCTCAGTATCTGGGGAATTGGTTCCAGGACCTCCCTTAA
ATATCAAAATGTGAGGATGCTCAAGCCCCTGATATAAAGTGGCACAGCATTTGTGTGTAACCTACAGACATCCTCCCATCTAC
AGCATCTCCTGATTACCTATAGTACCTAATACAGTGCAAATGCTATGTGAATAATTGTTATGCTGTATTGTTTAGGGAATAATGA
CAAGAAAAAAAGTCCGTATATGTTCAGTACCGATGCAACA

d

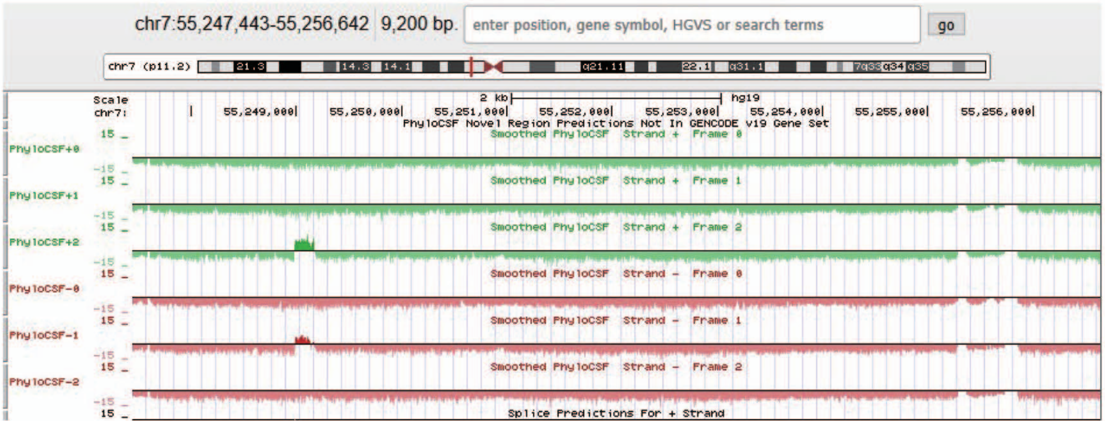

Figure S2

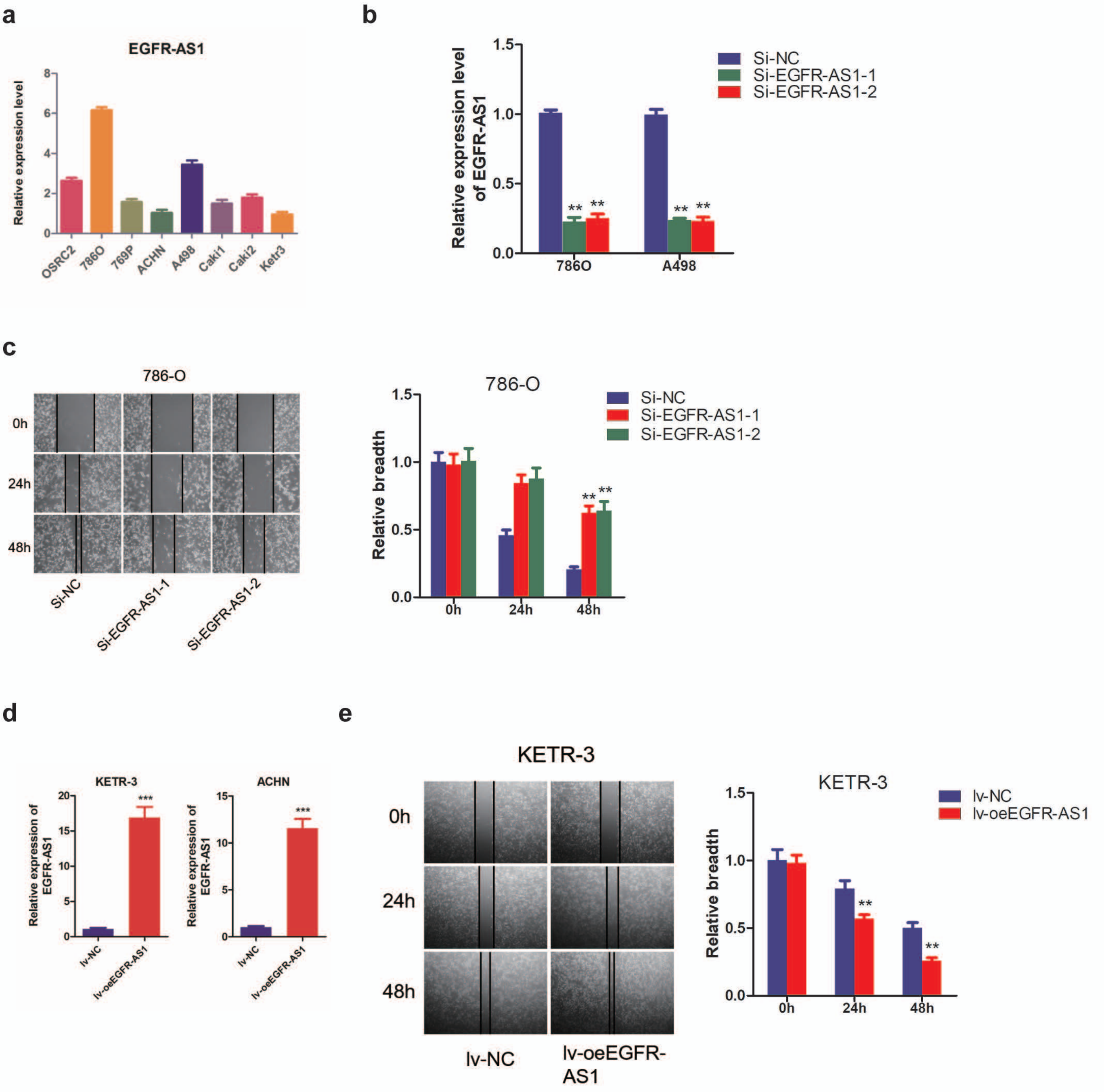
a
EGFR-AS1
Relative expression level
OSRC2
786O
769P
ACHN
A498
Caki1
Caki2
Ketr3
b
Relative expression level of EGFR-AS1
Si-NC
Si-EGFR-AS1-1
Si-EGFR-AS1-2
786O
A498
c
786-O
0h
24h
48h
Si-NC
Si-EGFR-AS1-1
Si-EGFR-AS1-2
Relative breadth
d
KETR-3
ACHN
Relative expression of EGFR-AS1
lv-NC
lv-oeEGFR-AS1
e
KETR-3
lv-NC
lv-oeEGFR-AS1
Relative breadth

Figure S3

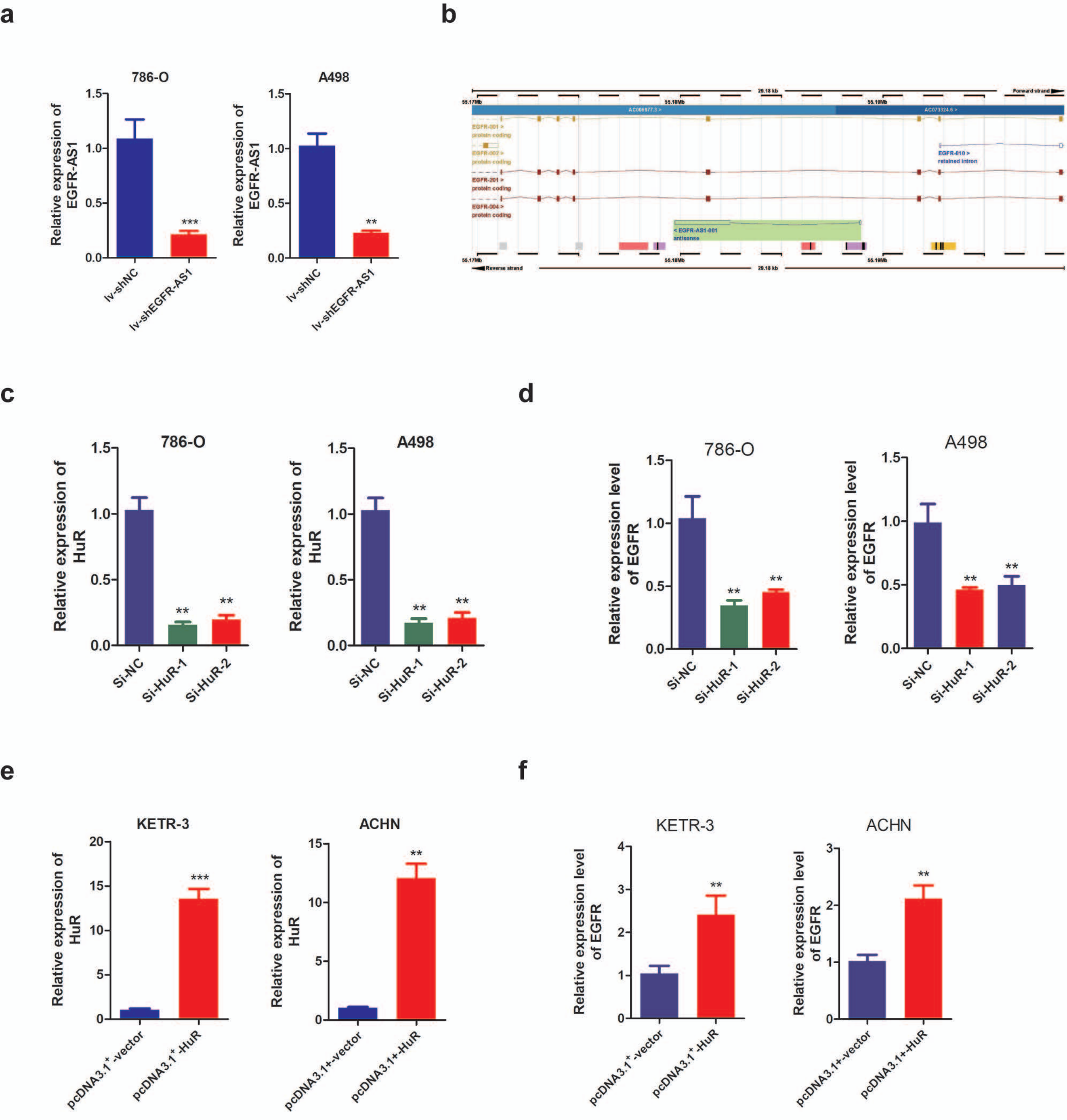

a
786-O
A498
Relative expression of EGFR-AS1
1.5
1.0
0.5
0.0
***
**
lv-shNC
lv-shEGFR-AS1
b
29.18 kb
Forward strand
55.17Mb
55.18Mb
55.19Mb
Reverse strand
EGFR-001 >
protein coding
EGFR-002 >
protein coding
EGFR-201 >
protein coding
EGFR-004 >
protein coding
EGFR-010 >
retained intron
< EGFR-AS1-001
antisense
c
786-O
A498
Relative expression of HuR
**
Si-NC
Si-HuR-1
Si-HuR-2
d
786-O
A498
Relative expression level of EGFR
**
Si-NC
Si-HuR-1
Si-HuR-2
e
KETR-3
ACHN
Relative expression of HuR
20
15
10
5
0
***
**
pcDNA3.1+-vector
pcDNA3.1+-HuR
f
KETR-3
ACHN
Relative expression level of EGFR
4
3
2
1
0
**
pcDNA3.1+-vector
pcDNA3.1+-HuR

Figure S4

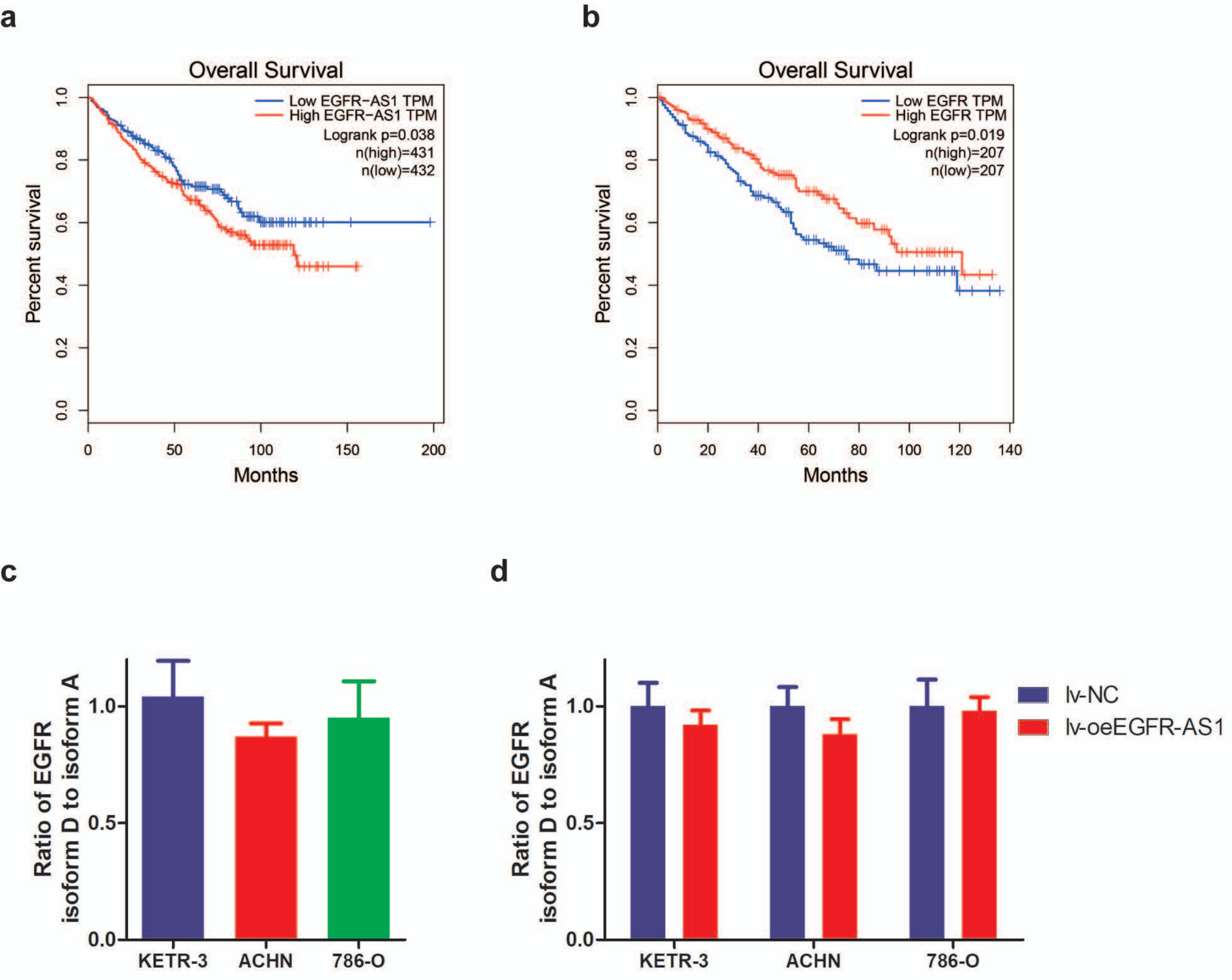
a
b
c
d
Overall Survival
Low EGFR−AS1 TPM
High EGFR−AS1 TPM
Logrank p=0.038
n(high)=431
n(low)=432
Percent survival
Months
Low EGFR TPM
High EGFR TPM
Logrank p=0.019
n(high)=207
n(low)=207
Ratio of EGFR isoform D to isoform A
KETR-3
ACHN
786-O
lv-NC
lv-oeEGFR-AS1
