# Supplementary material for: Long noncoding RNA EGFR-AS1 promotes cell growth and metastasis via affecting HuR mediated mRNA stability of EGFR in renal cancer
Source: Cell Death Dis. 2019 Feb 15;10(3):154. doi: 10.1038/s41419-019-1331-9 (PMC6377662; doi:10.1038/s41419-019-1331-9)
Supplement: Supplementary file 3 — Supplementary information [file 41419_2019_1331_MOESM3_ESM.docx]

**Supplementary information**

**Supplementary Table: Table S1.** Sequences of primers used for qRT-PCR in this study. **Table S2.** Sequences of primers used for siRNAs and plasmid construction in this study. **Table S3.** Mass spectrometry analysis of the proteins pulled down by EGFR-AS1 in 786-O cells. **Table S4.** The correlation analysis of EGFR-AS1 with EGFR isoforms in TCGA KIRC database.

**Supplementary Figure: Figure S1. a** lncRNAs in a large database analysis comparing RCC samples to paracancerous tissues. The results are shown in a Venn diagram. Those on the right were upregulated in the intersection of 5 datasets. The left presents those downregulated at the intersection of 5 datasets. **b** A23.1 and 10.4 expression between RCC samples and paired normal tissues were analyzed using qRT-PCR, respectively (n = 40). P < 0.05 by the Mann-Whitney U test. **c** The nucleotide sequence of full-length human EGFR-AS1 is 2747 bp long. **d** The codon substitution frequency scores (PyhloCSF) of EGFR-AS1.

**Figure S2. a** qRT-PCR analysis of EGFR-AS1 in RCC cell lines. **b** Relative expression of EGFR-AS1 in 786-O and A498 cells transfected with siRNAs compared with those in the control group. **c** Left: representative images of wound-healing assays performed in EGFR-AS1 overexpressing and NC KETR-3 cells photographed at 0, 24 and 48 hours after scratching. Scale bar = 800 μm. Right: the relative migration rates were assessed and calculated among the groups. **d** Relative expression of EGFR-AS1 in the lv-oeEGFR-AS1 lentivirus group compared to the lv-NC group in KETR-3 and ACHN cells. **e** Left: representative images of wound-healing assays performed when EGFR-AS1 was knocked down with siRNAs or the NC group in 786-O cells photographed at 0, 24 and 48 hours after scratching. Scale bar = 800 μm. Right: the relative migration rates were assessed and calculated among the groups.

**Figure S3.** **a** Relative expression of EGFR-AS1 in the lv-shEGFR-AS1 lentivirus group compared to the lv-shNC group in 786-O and A498 cells. **b** The diagrammatic sketch of EGFR-AS1 and EGFR in genome location. **c** Relative expression of HuR in 786-O and A498 cells transfected with siRNAs compared with those in the control group. **d** Relative expression of HuR in the HuR overexpressing group compared to control group in KETR-3 and ACHN cells. **e** Relative expression of EGFR at the mRNA level between HuR knockdown and control group RCC cell lines. **f** Relative expression of EGFR at the mRNA level between the HuR overexpression and control group RCC cell lines.

**Figure S4. a** Kaplan–Meier analysis of overall survival rate of RCC patients with high or low EGFR-AS1 expression in TCGA database (*P* = 0.038). **b** Kaplan–Meier analysis of overall survival rate of RCC patients with high or low EGFR expression in TCGA database (*P* = 0.019). **c** The relative ratio of EGFR isoform D to isoform A in KETR-3, ACHN and 786-O RCC cell lines. **d** The relative ratio of EGFR isoform D to isoform A in lv-oeEGFR-AS1 and lv-NC RCC cell lines.
